# Social relationships and mental wellbeing in autistic adolescent girls and boys: A longitudinal investigation

**DOI:** 10.1002/jcv2.70098

**Published:** 2026-03-04

**Authors:** Ellie Roberts, Eirini Flouri, Will Mandy

**Affiliations:** ^1^ Division of Biosciences University College London London UK; ^2^ Department of Psychology and Human Development Institute of Education University College London London UK; ^3^ Research Department of Clinical Educational, & Health Psychology University College London London UK

**Keywords:** adolescence, autism, gender, relationships, wellbeing

## Abstract

**Background:**

Previous studies have reported differences in levels of mental wellbeing between autistic and non‐autistic adolescents and between girls and boys. However, it is unclear to what extent being autistic or a particular gender influences mental wellbeing in adolescence. The importance of social relationships for mental wellbeing is well established, but it is unknown to what extent this may differ between autistic and non‐autistic adolescents and between autistic girls and boys.

**Methods:**

Data from the Millennium Cohort Study were used. Measures of social experience included social support and social alienation at age 14 (*N* = 11,056). Mental wellbeing was measured at age 17 (*N* = 10,034). Moderated regression analyses after multiple imputation were used to determine (i) whether autism diagnosis moderates the effect of social experience on mental wellbeing and (ii) whether the interaction between gender and autism diagnosis moderates the effect of social experience on mental wellbeing (*N* = 16,370).

**Results:**

In this nationally representative sample, levels of mental wellbeing are lower for autistic adolescents and for girls. Autistic girls had the lowest wellbeing, with large differences relative to non‐autistic boys (mean difference: −3.09; 95% CI: −4.565, −1.612) and non‐autistic girls (mean difference: −1.66; 95% CI: −3.13, −0.180). Autistic boys had lower wellbeing compared to non‐autistic boys (mean difference: −1.68; 95% CI: −2.54, −0.824) and non‐autistic girls (mean difference: −1.43; 95% CI: −1.69, −1.18). Autism does not moderate the effect of social experiences on mental wellbeing. No moderations were found on the effect of social experiences on mental wellbeing when all potential interactions are considered.

**Conclusion:**

Social support and social alienation during early adolescence equally impact the mental wellbeing of autistic and non‐autistic individuals at later adolescence. Considering the low mental wellbeing of autistic adolescents, there is a critical need to improve their social experiences.

## Key Points

**What's known?**

- Differences have been reported in the social experiences and mental wellbeing of adolescents based on autism diagnosis or gender.

**What's new?**

- Autistic girls have poorer mental wellbeing than all non‐autistic adolescents, while autistic boys only have poorer mental wellbeing compared to non‐autistic boys but not non‐autistic girls.
- Social support is equally beneficial to the mental wellbeing of autistic adolescents compared to non‐autistic adolescents, and social alienation equally detrimental.
- Neither social support or social alienation appear to differentially impact the mental wellbeing of adolescent girls and boys.

**What's relevant?**

- Improving autistic adolescents' social experiences should be a target intervention to address the lower mental wellbeing of autistic adolescents compared to non‐autistic adolescents.

## INTRODUCTION

Autism is a neurodevelopmental condition characterised by differences in social communication, sensory processing and restricted, repetitive behaviours (American Psychiatric Association, 2013). Autistic individuals experience higher rates of mental health problems compared to the non‐autistic population (Curnow et al., 2023). They also report lower levels of mental wellbeing, a related but separate construct to mental health (Patalay & Fitzsimons, 2016). Definitions of mental wellbeing are varied but generally involve the sustainable state of functioning well and feeling good (Huppert, 2009), with emphasis on thriving in one's environment, rather than simply being free of mental health symptoms (Huppert, 2014). Measurement is varied and includes consideration of positive/negative affect, autonomy, and satisfaction with personal life but also employment, income, and education (Najeeb & Quadt, 2024). Although mental health symptoms have been found to influence mental wellbeing (Oakley et al., 2021), associations between mental health and mental wellbeing are typically weak and the two constructs have distinct predictors (Patalay & Fitzsimons, 2016).

Autistic individuals report lower levels of mental wellbeing compared to non‐autistic individuals, and this has been observed in adolescents and young adults (Ridgway et al., 2024) as well as mature adults (Oakley et al., 2021). They have also highlighted the importance of reframing research to explore wellbeing rather than mental ill health (Najeeb & Quadt, 2024). As a result, there has been an increasing shift in autism research towards investigating wellbeing (Najeeb & Quadt, 2024; Pellicano & Heyworth, 2023). Despite this progress, there is a critical need for more research to investigate predictors and correlates of mental wellbeing in autistic people. To date, many factors have been associated with the wellbeing of autistic individuals, such as depression and anxiety, self‐empowering characteristics, community engagement, self‐identity, and relationships (Najeeb & Quadt, 2024). Of particular interest, but less well‐investigated, is the role of social support and relationships in determining wellbeing. Perceived social support and quality of relationships have a significant predictive role in the wellbeing of non‐autistic individuals (Siedlecki et al., 2014). This has also been observed for autistic adults (Maitland et al., 2021), in a cross‐sectional study. Differences in social communication and relationships are, by definition, a universal experience for autistic individuals, therefore it is of particular importance to understand how this may impact their wellbeing.

The social motivation theory of autism proposes that social interactions are less important to those who are autistic compared to those who are not (Chevallier et al., 2012). However, qualitative reports from autistic individuals suggest that social relationships are important for their wellbeing (Sedgewick et al., 2016). This includes incidental social contact with acquaintances and strangers that is, ‘weak ties’, as well as ‘close ties’ with family, friends, and partners (Pellicano & Heyworth, 2025). Indeed, autistic adults who report feeling connected with more groups have higher ratings of mental wellbeing (Maitland et al., 2021). Conversely, reduced quality of social interactions has been shown to increases the amount of negative affect experienced by autistic individuals (Dallman et al., 2022). Feeling accepted by non‐autistic individuals is associated with lower levels of mental distress in autistic people, partly due to a reduced need to ‘mask’ their natural autistic behaviour (Cage et al., 2018). At the same time, a frequent experience which likely contributes to differences in the social experience of autistic individuals is bullying, particularly in childhood and adolescence (Humphrey & Hebron, 2014). Therefore, there are contradicting ideas regarding how social experiences may differentially affect the mental wellbeing of autistic and non‐autistic individuals. This study will seek to contribute to this discussion about these phenomena in adolescence.

Adolescence is a particularly important developmental stage when considering the effects of social relationships on wellbeing. There is a gradual decrease in subjective wellbeing throughout adolescence for both non‐autistic and autistic adolescents (Begeer et al., 2017; González‐Carrasco et al., 2017). This is likely due to the complex biological, emotional and social development which occurs at this stage alongside an increase in negative affect (Grisanzio et al., 2023; Steinberg & Morris, 2001). At the same time, this is a period when social relationships, particularly peer relationships, become particularly important, a change attributed to structural and functional neural changes during adolescence (Blakemore, 2012). As both adolescence and autism influence social relationships and wellbeing, autism could raise additional complexities during adolescence and its associated social changes, due to pre‐existing social differences. It is, therefore, plausible that the effect of social difficulties on wellbeing may be larger in autistic compared to non‐autistic adolescents. Recently, Oakley et al. (2021) found that, while depression symptoms could explain reduced wellbeing across all ages of autistic individuals, there was an additional role of social communication difficulties in adolescents. Indeed, autistic adolescents report higher levels of loneliness compared to non‐autistic adolescents (Whitehouse et al., 2009).

It is also possible that the link between social relationships and wellbeing in autistic adolescents may vary by gender. Compared to boys, girls typically report lower levels of wellbeing in samples of the general population (González‐Carrasco et al., 2017; Yoon et al., 2023). Girls report lower wellbeing than boys at ages 11–12 years, with their level of wellbeing deteriorating year by year throughout adolescence, while boys' wellbeing remains relatively stable (Yoon et al., 2023). Girls are also found to view social relationships as more important and demonstrate differences to boys in a range of social behaviours (Rose & Rudolph, 2006). Rose and Rudolph (2006) propose an integrative peer‐socialisation model, whereby exposure to peers of the same sex contributes to sex‐typed peer relationship processes. These processes are hypothesised to influence the emotional and behavioural development of girls and boys. Differences in social experiences, such as understanding of friendships and activities with friends (Dean et al., 2017; Sedgewick et al., 2016), have also been observed between autistic girls and boys. Subsequently, the gender of autistic adolescents must also be considered when investigating the role of social relationships in mental wellbeing.

We have previously found that social experiences differ between adolescents (aged 14 years) based on gender and autism diagnosis (Roberts et al., 2025). For example, we observed that autistic adolescents feel less socially supported and more socially alienated than non‐autistic adolescents, and that girls feel more socially alienated than boys. Our previous study focused on identifying differences in social experiences at age 14. It remains uncertain as to whether these experiences have a role in predicting mental wellbeing in later adolescence and whether they can contribute to understandings of any differences in levels of wellbeing between autistic and non‐autistic individuals and between girls and boys.

### The present study

The evidence discussed suggests the role of social relationships on adolescent wellbeing may vary by autism, gender and their interaction. However, while the importance of social relationships to autistic individuals has been reported, it is not clear how this directly compares to that of non‐autistic individuals. Furthermore, social relationships appear to be more influential to the wellbeing of non‐autistic girls than boys, but it is unknown whether this observation is mirrored in autistic girls and boys. Most wellbeing autism research concentrates on mature adults (Najeeb & Quadt, 2024) despite the significant changes in wellbeing and relationships during adolescence. Furthermore, previous studies have typically only focused on experiences relating to either social support or social alienation and have not simultaneously investigated the importance of different social experiences. Finally, measures of wellbeing have focused on objective outcomes and less so on subjective or mental wellbeing, which typically is also modelled narrowly, for example, as self‐identity, autonomy, or acceptance (Najeeb & Quadt, 2024).

This study sought to fill these gaps using a population‐representative longitudinal cohort to understand to what extent autism, gender, or their interaction influence the effect of social experiences (age 14) on mental wellbeing (age 17) in adolescents. Direct comparisons were made between all groups: autistic girls, non‐autistic girls, autistic boys, and non‐autistic boys. The current study also directly compared the importance of different social experiences: social support and social alienation. These have been shown to be meaningfully distinct constructs in autistic and non‐autistic adolescents (Roberts et al., 2025).

## METHODS

### Sample

Participants were drawn from the Millenium Cohort Study (MCS), a birth cohort tracking the development of approximately 19,000 families of children born between 2000 and 2002 in England, Wales, Scotland and Northern Ireland (Joshi & Fitzsimons, 2016). In all, 11,056 were present for the measurement of relevant social experiences at age 14 years (Sweep 6) and 10,034 were present for the measurement of mental wellbeing at age 17 years (Sweep 7). Our study's analyses were carried out on the multiply imputed sample (*N* = 16,370) (those who had valid data on social experience or mental wellbeing and including the autistic sample) and the autistic sample (*N* = 498). Ethical approval for MCS was gained from NHS Multi‐Centre Ethics Committees, and informed consent was given before data collection took place.

### Measures

#### Autism

A single question asking whether the participant had ever been diagnosed with ‘autism, Asperger's syndrome or autistic spectrum disorder’ was administered at Sweeps 3 (age 5), 4 (age 7), 5 (age 11) and 6 (age 14). In line with previous work with MCS data, our autistic sample includes those whose parent or guardian responded ‘yes’ at least once and never ‘no’ after previously having responded ‘yes’ (e.g., Mandy et al., 2022). Parent‐reported clinician autism diagnoses have previously been found to be valid (Daniels et al., 2012). Further evidence for the validity of this method of case identification comes from the fact that: (i) the autism group have a similar male‐to‐female ratio (i.e., ∼4‐to‐1) (e.g., Hosozawa et al., 2020) and rates of ADHD prevalence (∼30%) (e.g., Mandy et al., 2022) as found in the wider autism literature (Lai et al., 2017; Loomes et al., 2017) and (ii) the autism group, compared to the non‐autistic group, show a range of mental health and social challenges that would be expected in an autistic sample (e.g., Hosozawa et al., 2020; Mandy et al., 2022; Roberts et al., 2025; Suominen et al., 2026).

#### Social experiences

Two factors from a previous factor analysis (Roberts et al., 2025) were used in this study, comprising measures of social support and social alienation, with constituent items measured at Sweep 6 (age 14). Social support included the items ‘have family and friends who make me feel supported and safe’, ‘feel like I have someone to turn to’, ‘keep worries to myself’ and ‘no one feels close’. The latter two were reverse‐coded prior to factor analysis. Social alienation comprised the two measures of bullying in Sweep 6: ‘often picked on/hurt by others’ and ‘bullied online’, and the two items ‘felt lonely’ and ‘felt nobody really loved me’ (see Table S1 for item measures).

#### Mental wellbeing

Mental wellbeing was measured once in MCS, at Sweep 7 (age 17), using the Warwick‐Edinburgh Mental Wellbeing Scale (WEMWBS) (Tennant et al., 2007). Different versions of the scale exist with subsets of the original 14 items. The scale administered in MCS was a seven‐item version which included the items: ‘I've been feeling optimistic about the future’, ‘I've been feeling useful’, ‘I've been feeling relaxed’, ‘I've been dealing with problems well’, ‘I've been thinking clearly’, ‘I've been feeling close to other people’ and ‘I've been able to make up my own mind about things’. Items were scored with responses of ‘none of the time’, ‘rarely’, ‘some of the time’, ‘often’ or ‘all of the time’.

#### Stratum

MCS sampling stratum recorded the area from which the MCS families were sampled, as MCS oversampled poor areas and ethnic minority families. In each UK country, families were divided into an advantaged and disadvantaged stratum. In England, there was also an ethnic minority stratum, which was assigned if at least 30% of the area's population fell into the categories of ‘Black’ (Black Caribbean, Black African or Black Other) or ‘Asian’ (Indian, Pakistani or Bangladeshi). The disadvantaged stratum comprised areas which did not meet the criteria for the ethnic minority stratum but represented the upper quartile (top 25%) of poorest areas as determined by the ward‐based Child Poverty Index. The advantaged stratum included areas which did not meet the criteria for the ethnic minority or disadvantaged stratum.

#### Confounders

Several confounders were considered in the analysis: ethnicity, parental education, family structure, number of siblings, hyperactivity, emotional problems, and verbal ability. Ethnicity was coded based on the UK Census classification: White, Black or Black British, Indian, Mixed, Pakistani, Bangladeshi, or Other Ethnic Group (including Chinese or Other). Parental education was recorded as the highest educational qualification obtained by the parental respondent, based on the UK's National Vocational Qualification (NVQ) classification, ranging from none (1) to the highest level (6). Family structure was measured as the number of parents present in the household (one or two). Number of siblings was an open‐ended measure. Hyperactivity and emotional problems were measured using the parent‐reported Strengths and Difficulties Questionnaire (SDQ) which has good reliability and validity in various population samples (Goodman, 2001). Verbal ability was used as a proxy for IQ and was assessed using the Word Task, which measured participants' understanding of word meaning. All confounders were measured at Sweep 6 (age 14).

### Analytic strategy

Analyses were conducted in STATA version 18. Descriptive statistics summarised the sample characteristics. Bivariate correlations using Pearson's correlation coefficients were conducted between the mental wellbeing, social experience (i.e., social support and social alienation), mental health, and verbal ability variables. Two‐way ANOVA was used to determine whether autism (diagnostic status), gender or their interaction predict mental wellbeing and social experience. Tukey's test was then used to conduct all pairwise comparisons between autistic girls, autistic boys, non‐autistic girls, and non‐autistic boys for mental wellbeing. Moderated regression analyses investigated whether diagnostic status moderates the effect of social experience on wellbeing in the whole sample. Multiple imputation by chained equations was used to handle missing data in the regression analyses. Diagnostic status was not imputed.

All analyses of tests of differences adjusted for the MCS sampling stratum. For the ANOVA, Tukey's test and regression analyses, three analytic models were used with different levels of adjustment. Stratum was adjusted for in all models. The unadjusted model only adjusted for stratum. Adjusted model 1 also included the confounders of ethnicity, family structure, number of siblings, and parental education. Adjusted model 2 added to adjusted model 1 scale scores on hyperactivity, emotional problems, and verbal ability. Social support and social alienation were mutually adjusted for in all models.

## RESULTS

### Descriptive statistics

Descriptive statistics for the sample can be seen in Table 1. The sample was relatively equally divided by sex. Most participants were of white ethnicity and were living in two‐parent households. The most common level of parental educational attainment was equivalent to NVQ Level 4 (certificate of higher education).

**TABLE 1:** Descriptive statistics of the sample (unweighted data).

Categorical variables		*N* (%)
Autism diagnosis		498 (3.0)
Sex		
Female		8015 (49.0)
Male		8355 (51.0)
Ethnicity		
White		9098 (79.5)
Mixed		543 (4.7)
Indian		309 (2.7)
Pakistani		584 (5.1)
Bangladeshi		254 (2.2)
Black Caribbean		120 (1.1)
Black African		227 (2.0)
Other ethnic group		314 (2.7)
Parent home structure		
Two parents		8826 (75.4)
Single parent		2884 (24.6)
Highest parental education		
NVQ level 1		1203 (6.3)
NVQ level 2		4696 (24.4)
NVQ level 3		2668 (13.9)
NVQ level 4		6379 (33.2)
NVQ level 5		2295 (11.9)
Stratum		
England—Advantaged		4615 (24.9)
England—Disadvantaged		4515 (24.4)
England—Ethnic		2393 (12.9)
Wales—Advantaged		831 (4.5)
Wales—Disadvantaged		1926 (10.4)
Scotland—Advantaged		1144 (6.2)
Scotland—Disadvantaged		1188 (6.4)
N. Ireland—Advantaged		721 (3.9)
N. Ireland—Disadvantaged		1199 (6.5)

Continuous variables	*N*	Mean (SD), range
Social support^a^	11,056	5.505 (0.921), 3–10
Social alienation^a^	11,056	−7.847 (2.638), ‐10‐4
WEMWBS	10,034	22.46 (4.08), 7–35
Number of siblings		
0	1613	1.55 (1.16), 0–10
1	5135	
2+	4963	
IQ (verbal ability)	10,900	7.06 (2.63), 0–19
SDQ hyperactivity	11,449	2.99 (2.40), 0–10
SDQ emotional problems	11,454	2.05 (2.14), 0–10

^a^ Total item scores for factors are presented in this table but factor scores are used in the analysis throughout this study.

### Bivariate correlations

Bivariate associations were calculated using Pearson's correlation coefficients for the whole sample and for the subsample with an autism diagnosis (see Table S2). Wellbeing correlated negatively with social alienation (whole sample *r* = −0.31, *p* < 0.001; autistic sample *r* = −0.28, *p* < 0.001) but positively with social support (whole sample *r* = 0.28, *p* < 0.001; autistic sample *r* = 0.27, *p* < 0.001). Emotional problems (whole sample *r* = −0.17, *p* < 0.001; autistic sample *r* = −0.15, *p* < 0.001) and hyperactivity (whole sample *r* = −0.10, *p* < 0.001; autistic sample *r* = −0.14, *p* < 0.001) were both negatively correlated with wellbeing. Social alienation and social support were negatively correlated (whole sample *r* = 0.63, *p* < 0.001; autistic sample *r* = 0.51, *p* < 0.001). Social alienation positively correlated with hyperactivity (whole sample *r* = 0.08, *p* < 0.001; autistic sample *r* = 0.14, *p* < 0.001) and emotional problems (whole sample *r* = −0.24, *p* < 0.001; autistic sample *r* = −0.33, *p* < 0.05). Social support demonstrated a negative correlation with hyperactivity only in the whole sample (*r* = −0.125, *p* < 0.001), and with emotional problems (whole sample *r* = −0.17, *p* < 0.001; autistic sample *r* = −0.15, *p* < 0.05). Emotional problems and hyperactivity were positively correlated (whole sample *r* = 0.36, *p* < 0.001; autistic sample *r* = 0.36, *p* < 0.001).

### Mental wellbeing by group

**FIGURE 1:**
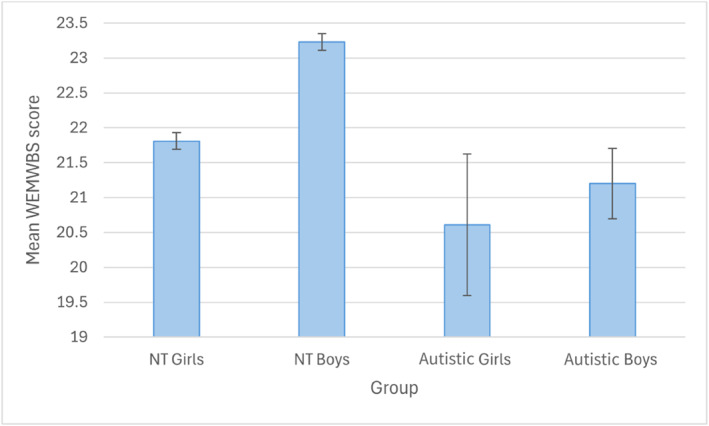
Bar graph demonstrating differences between mean WEMWBS score by gender and autism status with confidence intervals.

Mean values for mental wellbeing differed by group (Table 2; neurotypical (NT) refers to individuals who are non‐autistic). Autistic girls had the lowest mean wellbeing score, followed by autistic boys, neurotypical girls, and then neurotypical boys. Differences between groups have been visualised in Figure 1. Mean values for the social experience variables have been reported elsewhere (Roberts et al., 2025) (see also Table S3).

**TABLE 2:** Mean values for mental wellbeing by group (unweighted data).

Variable (Prob > *F*)	NT girls (*N* = 7904)		NT boys (*N* = 7968)		Autistic girls (*N* = 111)		Autistic boys (*N* = 387)	
	Mean	SD	Mean	SD	Mean	SD	Mean	SD
WEMWBS***	21.81 (*N* = 4693)	4.10	23.23 (*N* = 4276)	3.989	20.61 (*N* = 72)	4.40	21.20 (*N* = 228)	3.89

***p < 0.001.

### Two‐way ANOVA and Tukey's test

Results from two‐way ANOVA and Tukey's test for mental wellbeing can be seen in Table 3. Lower levels of wellbeing were reported by autistic individuals and girls. Autistic girls had lower levels of wellbeing than non‐autistic boys (Cohen's *d* = 0.66), representing the largest difference in wellbeing, as well as non‐autistic girls (Cohen's *d* = 0.29). Compared to non‐autistic boys, lower wellbeing was observed in non‐autistic girls (Cohen's *d* = 0.35) and autistic boys (Cohen's *d* = 0.51). Results from two‐way ANOVA and Tukey's test for the social experience variables have been reported elsewhere (Roberts et al., 2025) (see Table S4).

**TABLE 3:** Two‐way ANOVA and Tukey's test (complete data).

Mental wellbeing					
Predictor (*N* = 7018)	Partial SS	*df*		*F*	Prob > *F*
Autism diagnosis***	440.896	1		27.62	<0.0001
Gender (female)***	311.224	1		19.49	<0.0001
Autism* gender	0.090	1		0.01	0.9401
Adjusted (*N* = 6675)					
Autism diagnosis***	399.618	1		25.17	<0.0001
Gender (female)***	292.069	1		18.39	<0.0001
Autism* gender	0.020	1		0.00	0.9715
Adjusted 2 (*N* = 6237)					
Autism diagnosis	69.890	1		4.50	0.0339
Gender (female)***	215.219	1		13.86	0.0002
Autism* gender	2.554	1		0.16	0.6851

**Comparison**	**Contrast**	**SE**	***t***	***p* > [*t*]**	**95% CI**
NT girls versus NT boys***	−1.424	0.097	−14.67	<0.001	−1.673, −1.174
Autistic boys versus NT boys***	−1.691	0.319	−5.30	<0.001	−2.511, −0.871
Autistic girls versus NT boys***	−3.067	0.548	−5.59	<0.001	−4.476, −1.657
Autistic boys versus NT girls	−0.268	0.319	−0.84	0.836	−1.086, 0.551
Autistic girls versus NT girls*	−1.643	0.548	−3.00	0.014	−3.052, −0.235
Autistic girls versus Autistic boys	−1.376	0.627	−2.20	0.124	−2.986, 0.234
Adjusted					
NT girls versus NT boys***	−1.433	0.099	−14.42	<0.001	−1.688, −1.177
Autistic boys versus NT boys***	−1.680	0.333	−5.04	<0.001	−2.536, −0.824
Autistic girls versus NT boys***	−3.089	0.575	−5.38	<0.001	−4.565, −1.612
Autistic boys versus NT girls	−0.247	0.332	−0.74	0.880	−1.101, 0.607
Autistic girls versus NT girls*	−1.656	0.574	−2.88	0.021	−3.132, −0.180
Autistic girls versus Autistic boys	−1.409	0.655	−2.15	0.137	−3.093, 0.275
Adjusted 2					
NT girls versus NT boys***	−1.434	0.107	−13.41	<0.001	−1.709, −1.159
Autistic boys versus NT boys	−0.892	0.351	−2.54	0.054	−1.795, 0.011
Autistic girls versus NT boys**	−1.929	0.602	−3.21	0.007	−3.475, −0.383
Autistic boys versus NT girls	0.542	0.352	1.54	0.412	−0.361, 1.445
Autistic girls versus NT girls	−0.495	0.600	−0.82	0.843	−2.038, 1.048
Autistic girls versus Autistic boys	−1.037	0.677	−1.53	0.418	−2.777, 0.703

*p < 0.05; **p < 0.01; ***p < 0.001.

### Moderation analysis by diagnostic status

Results for regression models testing moderation by diagnostic status in the effect of social support and social alienation on wellbeing can be seen in Table 4. No moderation by diagnostic status was observed at any level of adjustment, either for social support or for social alienation.

**TABLE 4:** Moderation analysis by diagnostic status for the whole sample (*N* = 16,370).

	*b*	SE	95% CI
Social support (age 14) → mental wellbeing (age 17)			
Unadjusted model			
Social support***	1.349	0.054	1.241, 1.457
Autism diagnosis*	−0.561	0.234	−1.023, −0.099
Moderation	−0.073	0.254	−0.573, 0.427
Adjusted model 1			
Social support***	1.325	0.054	1.217, 1.434
Autism diagnosis***	−0.822	0.228	−1.272, −0.371
Moderation	−0.034	0.248	−0.522, 0.454
Adjusted model 2			
Social support***	1.224	0.054	1.117, 1.332
Autism diagnosis	−0.368	0.227	−0.816, 0.079
Moderation	−0.078	0.251	−0.572, 0.416
Social alienation (age 14) → mental wellbeing (age 17)			
Unadjusted model			
Social alienation***	−1.387	0.049	−1.485, −1.290
Autism diagnosis*	−0.584	0.238	−1.055, −0.113
Moderation	0.034	0.241	−0.442, 0.510
Adjusted model 1			
Social alienation***	−1.275	0.052	−1.380, −1.171
Autism diagnosis***	−0.777	0.233	−1.237, −0.317
Moderation	0.022	0.239	−0.449, 0.493
Adjusted model 2			
Social alienation***	−1.172	0.051	−1.273, −1.070
Autism diagnosis	−0.378	0.232	−0.837, 0.081
Moderation	0.089	0.236	−0.378, 0.555

*p < 0.05; ***p < 0.001.

### Moderation analysis by diagnostic status and gender

Results for moderated regression models taking into account the effects of interactions between social support/alienation, gender, and diagnostic status on mental wellbeing can be seen in Table 5. No moderating effects were found before or after adjustment.

**TABLE 5:** Moderation analysis by diagnostic status and gender for the whole sample (*N* = 16,370).

	*b*	SE	95% CI
Social support (age 14) → mental wellbeing (age 17)			
Unadjusted model			
Social support***	1.274	0.075	1.124, 1.423
Gender (female)***	−1.367	0.090	−1.546, −1.189
Autism diagnosis***	−0.955	0.282	−1.512, −0.399
Social support × gender	0.105	0.092	−0.078, 0.287
Social support × autism	−0.088	0.325	−0.730, 0.555
Autism × gender	0.382	0.495	−0.594, 1.358
Social support × autism × gender	0.241	0.621	−0.991, 1.474
Adjusted model 1			
Social support***	1.267	0.075	1.118, 1.417
Gender (female)***	−1.365	0.089	−1.542, −1.187
Autism diagnosis***	−0.941	0.282	−1.498, −0.385
Social support × gender	0.110	0.092	−0.073, 0.292
Social support × autism	−0.086	0.322	−0.722, 0.550
Autism × gender	0.382	0.492	−0.589, 1.354
Social support × autism × gender	0.250	0.623	−0.986, 1.485
Adjusted model 2			
Social support***	1.175	0.075	1.025, 1.325
Gender (female)***	−1.391	0.090	−1.571, −1.210
Autism diagnosis	−0.437	0.280	−0.989, 0.115
Social support × gender	0.094	0.092	−0.087, 0.276
Social support × autism	−0.169	0.328	−0.817, 0.480
Autism × gender	0.215	0.490	−0.751, 1.181
Social support × autism × gender	0.373	0.626	−0.870, 1.616
Social alienation (age 14) → mental wellbeing (age 17)			
Unadjusted model			
Social alienation***	−1.300	0.078	−1.456, −1.144
Gender (female)***	−0.916	0.096	−1.109, −0.724
Autism diagnosis***	−0.920	0.273	−1.458, −0.382
Social alienation × gender	0.037	0.093	−0.147, 0.222
Social alienation × autism	0.089	0.308	−0.518, 0.696
Autism × gender	0.572	0.497	−0.407, 1.551
Social alienation × autism × gender	−0.315	0.514	−1.330, 0.699
Adjusted model 1			
Social alienation***	−1.296	0.078	−1.452, −1.139
Gender (female)***	−0.917	0.096	−1.110, −0.725
Autism diagnosis***	−0.913	0.274	−1.454, −0.371
Social alienation × gender	0.037	0.093	−0.148, 0.222
Social alienation × autism	0.089	0.305	−0.514, 0.691
Autism × gender	0.564	0.492	−0.404, 1.532
Social alienation × autism × gender	−0.304	0.512	−1.315, 0.706
Adjusted model 2			
Social alienation***	−1.192	0.077	−1.346, −1.039
Gender (female)***	−1.008	0.097	−1.202, −0.814
Autism diagnosis	−0.449	0.275	−0.992, 0.094
Social alienation × gender	0.036	0.093	−0.148, 0.219
Social alienation × autism	0.159	0.306	−0.446, 0.763
Autism × gender	0.324	0.490	−0.641, 1.288
Social alienation × autism × gender	−0.249	0.514	−1.265, 0.766

***p < 0.001.

## DISCUSSION

The current study sought to compare mental wellbeing by both autism diagnostic status and gender, and to investigate whether the impact of social experiences (social support and social alienation) at age 14 on wellbeing at age 17 depends on diagnostic status, gender and their interaction. Ratings of wellbeing varied between groups. Autistic individuals and girls reported the lowest levels of mental wellbeing. Diagnostic status did not moderate the effect of social support or social alienation, aged 14 years, on wellbeing at 17 years, and neither did its interaction with gender. These findings demonstrate differences in the mental wellbeing of adolescents by both gender and diagnostic status, and that social experiences at age 14 are no less predictive of wellbeing at age 17 in autistic adolescents compared to non‐autistic adolescents.

The finding that autistic adolescents experience lower levels of mental wellbeing reflects those of previous studies (Oakley et al., 2021; Ridgway et al., 2024). This has been proposed to arise through multiple factors, such as poorer mental health, social isolation and loneliness, bullying, and unsuitable environments (Maitland et al., 2021; Najeeb & Quadt, 2024; O'Connor et al., 2024). The current findings also mirrored those of previous studies regarding the lower levels of mental wellbeing in non‐autistic girls compared to non‐autistic boys (González‐Carrasco et al., 2017; Yoon et al., 2023). However, the current study also demonstrates that autistic girls specifically have poorer mental wellbeing than all non‐autistic adolescents, while autistic boys only have poorer mental wellbeing compared to non‐autistic boys but not non‐autistic girls. The difficulties with wellbeing were especially pronounced in autistic girls, who are likely at particular risk of experiencing low levels of mental wellbeing in adolescence. This may be due to factors more prevalent in autistic girls, such as masking, relational bullying, and mental health problems (Cook et al., 2021; O'Connor et al., 2024; Sedgewick et al., 2019). Non‐autistic girls report similar social experiences as autistic girls (Sedgewick et al., 2019), which may contribute to lower levels of wellbeing in girls in general, however autistic girls may especially struggle with these challenges. Begeer et al. (2017) found that gender did not influence the subjective wellbeing of autistic or non‐autistic children, measured by parent report. Our contrasting findings may reflect the older age of our sample and/or the fact that wellbeing was self‐reported. We argue that self‐report measures of wellbeing, a subjective construct, are likely more valid (Gough Kenyon et al., 2021).

Results from the second adjusted model, which additionally controlled for emotional problems and hyperactivity, revealed that only differences in wellbeing between autistic girls and non‐autistic boys and between non‐autistic girls and non‐autistic boys remained, with autistic girls continuing to experience lower levels of wellbeing in both comparisons. On the other hand, differences between autistic girls and non‐autistic girls and between autistic boys and non‐autistic boys no longer remained. This suggests that within‐gender differences in wellbeing by autism status may be partly explained by emotional problems and hyperactivity, which are more prevalent in autistic individuals. However, gender differences appear to persist beyond this additional consideration, indicating that gender differences in wellbeing cannot be explained by differences in emotional and behavioural difficulties. Alternative mechanisms may include more direct pathways from cultural factors, such as differences in social standards for girls and boys (González‐Carrasco et al., 2017).

Feeling socially supported or socially alienated were no less influential in the mental wellbeing of autistic adolescents compared to non‐autistic adolescents. This challenges the belief that social relationships are less important to autistic individuals, as proposed by the social motivation theory of autism, which is described as an ‘extreme case of diminished social motivation’ (Chevallier et al., 2012). Observations such as lacking close friends, deriving less pleasure from friendships, being less responsive, and diminished eye contact have been interpreted to represent a lack of interest in social interaction and relationships (Chevallier et al., 2012). The current findings do not support this notion but rather suggest that social difficulties are equally impactful for autistic and for non‐autistic adolescents. This accords with multiple reports from autistic people themselves, that they value social relationships and are sensitive to difficulties in these (Chapman et al., 2022; Sedgewick et al., 2016). Autistic individuals may have different support needs due to social communication differences, but, according to our findings, social support is equally beneficial to their mental wellbeing, and social alienation equally detrimental. This highlights a pressing need for intervention to improve the social experiences of autistic adolescents, who demonstrate lower wellbeing and feel less socially supported and more socially alienated.

Somewhat unexpectedly, no interactions with gender were found regarding the effects of social experiences on mental wellbeing. Observations of non‐autistic adolescents suggest that social relationships are more important for the mental wellbeing of girls than boys (Johansen et al., 2021; Milner et al., 2016). This has also been suggested in qualitative reports with autistic women (Sedgewick et al., 2019). This discrepancy with the findings of the current study could be due to different measures of social support and mental wellbeing, as well as statistical power. Further research is required to explore a range of social experiences and how these may affect the wellbeing of autistic girls and boys.

These findings must be considered with the limitations of the study. Mental health was parent‐reported, as only the parent‐reported version of the SDQ was administered in Sweep 6. There was also no measure of general IQ at Sweep 6, so a measure of verbal ability was used instead (Smith et al., 2005). While this study demonstrates an association between social experiences at age 14 and mental wellbeing at age 17, it would be useful to model this relationship more comprehensively over time. Future research should seek to understand whether social experiences during early adolescence alone can predict mental wellbeing during later adolescence, or whether this is more due to an ongoing effect which may also be bidirectional. Definitions of mental wellbeing may also differ for different groups. Autistic individuals may place more emphasis on feelings of acceptance, competence, and identity when defining wellbeing (Featherstone et al., 2023), with a recent co‐produced measure of life quality highlighting the importance of identity, sensory processing, and healthcare (McConachie et al., 2018). Furthermore, different types of social relationships, not captured in this paper, may differentially impact individuals. For example, Luo et al. (2017) found that same‐sex friendships are more important to girls, while teacher‐student relationships were more important to boys. Future studies may wish to take into account these individual differences when investigating the effects of social experiences on mental wellbeing by autism, gender and their interaction. It is also of importance for further research to investigate the current study's research questions with regards to mental health, including the relationship between mental health and wellbeing, as mental wellbeing and mental health appear to demonstrate different correlates (Patalay & Fitzsimons, 2016).

## CONCLUSION

In this population‐representative cohort, autistic adolescents, compared to those who are not autistic, and girls, compared to boys, report lower levels of mental wellbeing. The effects of social support and social alienation at age 14 on mental wellbeing at age 17 are equivalent for autistic and non‐autistic boys and girls, highlighting the great need to address low wellbeing in autistic adolescents on the cusp of adulthood and facilitate healthy social relationships in all adolescents. Contrary to what some may have expected therefore, social relationships appear to have a significant effect on the wellbeing of autistic young people, an important conclusion. Future research should seek to model social relationships and their effects over time during adolescence and consider greater specificity in social interactions and definitions of wellbeing for different groups.

## AUTHOR CONTRIBUTIONS

**Ellie Roberts**: Conceptualization; formal analysis; investigation; methodology; visualization; writing—original draft. **Eirini Flouri**: Conceptualization; funding acquisition; methodology; supervision; writing—review and editing. **Will Mandy**: Conceptualization; funding acquisition; methodology; supervision; writing—review and editing.

## CONFLICT OF INTEREST STATEMENT

The authors declare no conflicts of interest.

## ETHICAL CONSIDERATIONS

This study uses secondary data analysis of the Millennium Cohort Study (MCS) data. The MCS data is publicly available from the UK Data Service, under licence, and its use does not require additional ethics approval; the primary data collection and public availability of data, for each survey wave, was subject to Multi‐Centre Ethics Committees led by the National Health Service in the United Kingdom. For more information on the MCS ethics approvals and related processes, consult for example, the UCL Institute of Education ‘Ethical review and consent’ (2012) document, available online: https://cls.ucl.ac.uk/wp‐content/uploads/2017/07/MCS‐Ethical‐review‐and‐consent‐Shepherd‐P‐November‐2012.pdf.

## Supporting information

Tables S1–S4

## Data Availability

Data are available in a public, open access repository. UK Data Service. University of London, Institute of Education, Centre for Longitudinal Studies. (2022). Millennium Cohort Study: First Survey, 2001–2003. 14th Edition. SN: 4683, DOI: http://doi.org/10.5255/UKDA‐SN‐4683‐6.
