# Supplementary material for: Social relationships and mental wellbeing in autistic adolescent girls and boys: A longitudinal investigation
Source: JCPP Adv. 2026 Mar 4:e70098. Online ahead of print. doi: 10.1002/jcv2.70098 (PMC13339046; doi:10.1002/jcv2.70098)
Supplement: Supplementary file 1 — Tables S1–S4 [file JCV2-9999-e70098-s001.docx]

**Social relationships and mental wellbeing in autistic adolescent girls and boys: a longitudinal investigation**

**Supporting Information**

**Table S1.** Constituent items for social support and social alienation factors

| **Social support** | |
| --- | --- |
| **Items** | **Scoring** |
| “I have family and friends who help me feel safe, secure and happy” “There is someone I trust whom I would turn to if I had problems”  “There is no one I feel close to”* | Very true Partly true Not true at all |
| “What do you do if you are worried about something? (Keep it to myself)”* | Yes No |
| **Social alienation** | |
| **Items** | **Scoring** |
| “I felt lonely”  “I thought nobody really loved me” | Not true  Sometimes  True |
| “How often do other children hurt or pick on you?” | Most days  About once a week  About once a month  Every few months  Less often  Never |
| “How often do other children bully you online?” | Most days  About once a week  About once a month  Every few months  Less often  Never |

*Reverse coded prior to factor analysis

**Table S2.** Bivariate correlations between the main continuous variables for the whole sample and autistic sample

| **Whole sample (N = 16,370)** | | | | | | |
| --- | --- | --- | --- | --- | --- | --- |
|  | Wellbeing | Alien | Support | Emotion | Hyper | Verbal |
| Wellbeing | 1 |  |  |  |  |  |
| Alien | -.308*** | 1 |  |  |  |  |
| Support | .276*** | -.631*** | 1 |  |  |  |
| Emotion | -.168*** | .244*** | -.166*** | 1 |  |  |
| Hyper | -.098*** | .084*** | -.125*** | .364*** | 1 |  |
| Verbal | .005 | .022* | .014 | -.110*** | -.216*** | 1 |
| **Autistic sample (N = 498)** | | | | | | |
|  | Wellbeing | Alien | Support | Emotion | Hyper | Verbal |
| Wellbeing | 1 |  |  |  |  |  |
| Alien | -.284*** | 1 |  |  |  |  |
| Support | .267*** | -.508*** | 1 |  |  |  |
| Emotion | -.147* | .326*** | -.146* | 1 |  |  |
| Hyper | -.142* | .136* | -.078 | .356* | 1 |  |
| Verbal | .017 | -.047 | .130* | -.002 | -.227*** | 1 |

Wellbeing = Mental wellbeing (age 17); Alien = Social alienation (age 14); Support = Social support (age 14); Emotion = Emotional problems; Hyper = Hyperactivity; Verbal = Verbal Ability, *p<.05; ***p<.001

**Table S3.** Group differences in social experiences (unweighted data)

| **Variable (Prob>F)** | **NT Girls (N = 7,904)** | | **NT Boys (N = 7,968)** | | **Autistic Girls (N = 111)** | | **Autistic Boys (N = 387)** | |
| --- | --- | --- | --- | --- | --- | --- | --- | --- |
|  | Mean | SD | Mean | SD | Mean | SD | Mean | SD |
| **Social support***** | -.010 N = 5,550 | .868 | .030 N = 5,180 | .790 | -.281 N = 76 | .932 | -.325 N = 250 | .979 |
| **Social alienation***** | .180 N = 5,550 | 1.011 | -.213 N = 5,180 | .730 | .824  N = 76 | 1.08 | .159 N = 250 | .888 |

***p<.001

**Table S4.** Two-way ANOVA and Tukey’s test for social experience variables (complete cases)

| **Social support factor** | | | | | |
| --- | --- | --- | --- | --- | --- |
| **Predictor** (N = 8,264) | **Partial SS** | **df** | | **F** | **Prob>F** |
| Autism diagnosis*** | 11.247 | 1 | | 16.44 | 0.0001 |
| Gender (Female) | .000 | 1 | | 0.00 | 0.9882 |
| Autism * Gender | .199 | 1 | | 0.29 | 0.5896 |
| **Adjusted** (N = 7,916) | | | | | |
| Autism diagnosis*** | 8.637 | 1 | | 12.86 | 0.0003 |
| Gender (Female) | .030 | 1 | | 0.05 | 0.8318 |
| Autism * Gender | .060 | 1 | | 0.09 | 0.7658 |
| **Adjusted 2** (N = 7,372) | | | | | |
| Autism diagnosis | .199 | 1 | | 0.30 | 0.5809 |
| Gender (Female) | .129 | 1 | | 0.20 | 0.6566 |
| Autism * Gender | .421 | 1 | | 0.64 | 0.4226 |
| **Comparison** | **Contrast** | **SE** | **t** | **P>[t]** | **95% CI** |
| NT Girls vs NT Boys | .034 | .018 | 1.83 | 0.259 | -.014, .081 |
| Autistic Boys vs NT Boys*** | .296 | .063 | 4.67 | 0.000 | .133, .460 |
| Autistic Girls vs NT Boys | .261 | .112 | 2.32 | 0.093 | -.028, .549 |
| Autistic Boys vs NT Girls*** | .263 | .063 | 4.14 | 0.000 | .100, .426 |
| Autistic Girls vs NT Girls | .227 | .112 | 2.02 | 0.181 | -.062, .516 |
| Autistic Girls vs Autistic Boys | -.036 | .128 | -0.28 | 0.992 | -.364, .292 |
| **Adjusted** | | | | | |
| NT Girls vs NT Boys | .031 | .019 | 1.67 | 0.338 | -.017, .079 |
| Autistic Boys vs NT Boys*** | .268 | .064 | 4.18 | 0.000 | .103, .432 |
| Autistic Girls vs NT Boys | .247 | .113 | 2.19 | 0.125 | -.042, .536 |
| Autistic Boys vs NT Girls*** | .237 | .064 | 3.70 | 0.001 | .072, .401 |
| Autistic Girls vs NT Girls | .216 | .113 | 1.92 | 0.222 | -.074, .505 |
| Autistic Girls vs Autistic Boys | -.021 | .128 | -0.16 | 0.998 | -.350, .308 |
| **Adjusted 2** | | | | | |
| NT Girls vs NT Boys | .022 | .020 | 1.11 | 0.683 | -.029, .074 |
| Autistic Boys vs NT Boys | .105 | .067 | 1.57 | 0.398 | -.067, .028 |
| Autistic Girls vs NT Boys | .006 | .117 | 0.05 | 1.000 | -.293, .306 |
| Autistic Boys vs NT Girls | .083 | .067 | 1.23 | 0.607 | -.090, .256 |
| Autistic Girls vs NT Girls | -.016 | .117 | -0.14 | 0.999 | -.315, .284 |
| Autistic Girls vs Autistic Boys | -.099 | .131 | -0.75 | 0.875 | -.435, .238 |
| **Social alienation factor** | | | | | |
| **Predictor** (N = 8,264) | **Partial SS** | **df** | | **F** | **Prob>F** |
| Autism diagnosis*** | 26.322 | 1 | | 33.73 | 0.0000 |
| Gender (Female)*** | 41.017 | 1 | | 52.56 | 0.0000 |
| Autism * Gender | 2.151 | 1 | | 2.76 | 0.0969 |
| **Adjusted** (N = 7,916) | | | | | |
| Autism diagnosis*** | 22.642 | 1 | | 29.14 | 0.0000 |
| Gender (Female)*** | 37.834 | 1 | | 48.70 | 0.0000 |
| Autism * Gender | 1.823 | 1 | | 2.35 | 0.1256 |
| **Adjusted 2** (N = 7,372) | | | | | |
| Autism diagnosis | 1.723 | 1 | | 2.31 | 0.1282 |
| Gender (Female)*** | 26.236 | 1 | | 35.25 | 0.0000 |
| Autism * Gender | .746 | 1 | | 1.00 | 0.3169 |
| **Comparison** | **Contrast** | **SE** | **t** | **P>[t]** | **95% CI** |
| NT Girls vs NT Boys*** | .385 | .020 | 19.51 | 0.000 | .334, .436 |
| Autistic Boys vs NT Boys*** | .286 | .068 | 4.22 | 0.000 | .112, .460 |
| Autistic Girls vs NT Boys*** | .900 | .120 | 7.50 | 0.000 | .591, 1.208 |
| Autistic Boys vs NT Girls | -.099 | .068 | -1.46 | 0.462 | -.273, .075 |
| Autistic Girls vs NT Girls*** | .515 | .120 | 4.29 | 0.000 | .206, .823 |
| Autistic Girls vs Autistic Boys*** | .614 | .136 | 4.50 | 0.000 | . 263, .964 |
| **Adjusted** | | | | | |
| NT Girls vs NT Boys*** | .382 | .020 | 18.97 | 0.000 | .330, .434 |
| Autistic Boys vs NT Boys*** | .272 | .070 | 3.91 | 0.001 | .094, .451 |
| Autistic Girls vs NT Boys*** | .870 | .122 | 7.12 | 0.000 | .556, 1.183 |
| Autistic Boys vs NT Girls | -.110 | .070 | -1.58 | 0.392 | -.288, .069 |
| Autistic Girls vs NT Girls*** | .487 | .122 | 3.99 | 0.000 | .174, .801 |
| Autistic Girls vs Autistic Boys*** | .597 | .139 | 4.30 | 0.000 | .240, .954 |
| **Adjusted 2** | | | | | |
| NT Girls vs NT Boys*** | .354 | .021 | 16.52 | 0.000 | .299, .409 |
| Autistic Boys vs NT Boys | .045 | .072 | 0.63 | 0.922 | -.139, .229 |
| Autistic Girls vs NT Boys*** | .545 | .124 | 4.38 | 0.000 | .225, .864 |
| Autistic Boys vs NT Girls*** | -.309 | .072 | -4.30 | 0.000 | -.493, -.124 |
| Autistic Girls vs NT Girls | .191 | .124 | 1.54 | 0.415 | -.128, .510 |
| Autistic Girls vs Autistic Boys** | .499 | .140 | 3.58 | 0.002 | .141, .858 |

**p<.01; ***p<.001
